# Serum renin and major adverse kidney events in critically ill patients: a multicenter prospective study

**DOI:** 10.1186/s13054-021-03725-z

**Published:** 2021-08-14

**Authors:** Alexander H. Flannery, Victor Ortiz-Soriano, Xilong Li, Fabiola G. Gianella, Robert D. Toto, Orson W. Moe, Prasad Devarajan, Stuart L. Goldstein, Javier A. Neyra

**Affiliations:** 1 Department of Pharmacy Practice and Science, University of Kentucky College of Pharmacy, Lexington, KY USA; 2 Department of Pharmacy Services, University of Kentucky HealthCare, Lexington, KY USA; 3 Department of Internal Medicine, Division of Nephrology, Bone, and Mineral Metabolism, University of Kentucky College of Medicine, Lexington, KY 40536 USA; 4 Department of Population and Data Sciences, University of Texas Southwestern Medical Center, Dallas, TX USA; 5 Charles and Jane Pak Center for Mineral Metabolism and Clinical Research, University of Texas Southwestern Medical Center, Dallas, TX USA; 6 Department of Internal Medicine, Division of Nephrology, University of Texas Southwestern Medical Center, Dallas, TX USA; 7 Center for Acute Care Nephrology, Cincinnati Children’s Hospital Medical Center, University of Cincinnati School of Medicine, Cincinnati, OH USA

**Keywords:** Renin, Acute kidney injury, Major adverse kidney events, Critical illness, Biomarker, Renin angiotensin system

## Abstract

**Background:**

Preliminary studies have suggested that the renin-angiotensin system is activated in critical illness and associated with mortality and kidney outcomes. We sought to assess in a larger, multicenter study the relationship between serum renin and Major Adverse Kidney Events (MAKE) in intensive care unit (ICU) patients.

**Methods:**

Prospective, multicenter study at two institutions of patients with and without acute kidney injury (AKI). Blood samples were collected for renin measurement a median of 2 days into the index ICU admission and 5–7 days later. The primary outcome was MAKE at hospital discharge, a composite of mortality, kidney replacement therapy, or reduced estimated glomerular filtration rate to ≤ 75% of baseline.

**Results:**

Patients in the highest renin tertile were more severely ill overall, including more AKI, vasopressor-dependence, and severity of illness. MAKE were significantly greater in the highest renin tertile compared to the first and second tertiles. In multivariable logistic regression, this initial measurement of renin remained significantly associated with both MAKE as well as the individual component of mortality. The association of renin with MAKE in survivors was not statistically significant. Renin measurements at the second time point were also higher in patients with MAKE. The trajectory of the renin measurements between time 1 and 2 was distinct when comparing death versus survival, but not when comparing MAKE versus those without.

**Conclusions:**

In a broad cohort of critically ill patients, serum renin measured early in the ICU admission is associated with MAKE at discharge, particularly mortality.

**Supplementary Information:**

The online version contains supplementary material available at 10.1186/s13054-021-03725-z.

## Background

The renin-angiotensin system (RAS) is increasingly recognized to be perturbed during critical illness. The complexity of this system, including circulatory, tissue-level, and intra-cellular actions, as well as both traditional and counter-regulatory axes, makes it difficult to simply classify the RAS as activated or insufficiently activated in critical illness; rather, it is dysfunctional [1]. RAS peptide analysis in vasodilatory shock [2] and acute respiratory distress syndrome [3] have strongly suggested a state of reduced angiotensin converting enzyme (ACE) and ACE2 function, respectively, which influences both the traditional and counter-regulatory axes of the RAS. Furthermore, reduced ACE and ACE2 function are associated with acute kidney injury (AKI) [4] and mortality [3], respectively, in critically ill patients. Biochemical evaluation of the RAS requires careful biospecimen collection and sophisticated analytical techniques [5]. However, upstream in the complex milieu of RAS peptide metabolism, renin plays a rate-limiting step in the conversion of angiotensinogen to angiotensin I, and is a far more readily available lab test for clinicians to consider at the bedside.

Recently, a single center study showed that plasma renin outperformed lactate in prognostic value for intensive care unit (ICU) mortality [6]. Subsequently, a small, single center study found no association of plasma renin with mortality, but did note plasma renin to be much higher with worsening AKI [7]. Given the potential of renin to serve as a novel biomarker for adverse kidney outcomes and mortality in critically ill patients, we conducted a multicenter prospective study to examine the association of serum renin with mortality and adverse kidney events in a larger and heterogeneous cohort of critically ill adult patients.

## Methods

This was a prospective, multicenter study of ICU patients from two academic medical centers. Patients were recruited sequentially from each site: University of Texas Southwestern (2015–2017) and the University of Kentucky (2017–2019). Adult patients ≥ 18 years old admitted to surgical, medical, or cardiac ICUs with a documented baseline estimated glomerular filtration rate (eGFR) ≥ 60 mL/min/1.73 m^2^ were eligible for inclusion. eGFR was calculated using the most recent outpatient serum creatinine in the 6 months prior to admission using the CKD-Epidemiology Collaboration equation [8]. Patients with end stage kidney disease (ESKD), evidence of AKI prior to ICU admission, uroepithelial tumors, or prior solid organ transplant were excluded.

The study purposefully included ICU patients with incident AKI based on Kidney Disease: Improving Global Outcomes (KDIGO) serum creatinine or urine output stage ≥ 2 criteria and ICU patients without AKI [9]. Blood samples were collected within the first 3 days of ICU admission (~ 24 h after AKI diagnosis for those with AKI). A second set of blood samples were collected 5–7 days following the initial collection. Demographic and comorbidity data were collected from the patient’s medical record for each enrolled patient. ICU-centric therapies were assessed, including the need for mechanical ventilation and vasopressor support. Patients’ comorbidities and severity of illness were further assessed with the Charlson Comorbidity Index [10] and Acute Physiology and Chronic Health Evaluation (APACHE) II score, respectively [11].

The primary outcome of the study was a major adverse kidney event (MAKE) by hospital discharge, defined as death, kidney replacement therapy (KRT) dependence at hospital discharge, or a reduction in eGFR to ≤ 75% of baseline at hospital discharge [12]. Each sub-component of the primary composite outcome was also assessed separately. Additional secondary outcomes included need for inpatient KRT, duration of mechanical ventilation, and ICU and hospital length of stay.

The study was approved by the institutional review boards at both participating centers. Patients, or their legally authorized representatives, provided written, informed consent for participation in the study.

### Laboratory analysis

Serum samples were processed within 45–60 min of collection, centrifuged at 1000*g* at 4 °C for 10 min, and stored at − 80 °C. Renin measurements were obtained using the active renin ELISA kit from DRG Diagnostics (Kit Reference ID: EIA-5125). The assay dynamic range is 0.80–128.0 pg/ml. The intra-assay CV is 2.4% and the inter-assay CV is 3.7%. The expected normal values in the serum or plasma range from 2.5 to 49.3 pg/ml. Neutrophil gelatinase-associated lipocalin (NGAL) measurements were obtained on a Siemens BNII using a particle-enhanced turbidimetric immunoassay from BioPorto (The NGAL Test, Kit Reference ID: ST001). Most samples (> 90%) were measured on the first freeze–thaw cycle. All other laboratory data were extracted from routine measurements available in the electronic health record.

### Statistical analysis

The first timepoint of renin measurements were assessed in tertiles. Medians and interquartile range and frequency (proportions) were used to provide descriptive statistics for the cohort. Comparisons among groups were done with Kruskal–Wallis (3 groups) or Wilcoxon rank-sum (2 groups) for continuous data and chi-square test for categorical data.

For the primary outcome of MAKE, a logistic regression model was constructed using renin tertiles as the independent variable of interest. Additional variables to include in the model were identified by the study group based upon their hypothesized ability to influence MAKE and according to imbalances in clinical parameters among renin tertiles that could potentially influence outcome. Given prior data demonstrating a strong association of serum NGAL with MAKE, including NGAL outperforming serum creatinine [13, 14], we used NGAL to adjust for the presence and severity of kidney injury. NGAL (and renin in the sensitivity analysis) were log-transformed due to the skewness of the data. Additional variables included in the model were: age, sex, race, baseline eGFR, Charlson Comorbidity Index, ICU type, study site, and non-renal APACHE II. In a pre-planned sensitivity analysis, renin was subsequently analyzed as a continuous variable in the logistic regression model for MAKE. Clinical parameters that were otherwise captured in scoring systems such as the Charlson Comorbidity Index and APACHE II were not included in order to avoid collinearity, which was assessed in the final models using variance inflation factors. When analyzing individual components of the MAKE outcome with logistic regression, we used backward stepwise regression (*p* < 0.2 threshold) given that we anticipated the reduced number of events to not accommodate the number of covariates used in the primary model. Finally, a mixed linear model was used to examine repeated measures analysis of serum renin levels between the two time points and their association with MAKE and the individual component of mortality.

A two-sided *p* value ≤ 0.05 was considered statistically significant. Stata (StataCorp. 2019. Stata Statistical Software: Release 16. College Station, TX: StataCorp LLC) and SAS 9.4 (SAS Institute, Cary, NC) were used for all statistical analyses.

## Results

From January 2015 to September 2019, 280 patients were enrolled in this prospective study with available blood samples for analysis. The median time from ICU admission to initial sample collection in the cohort was 2 (1–3) days. Baseline patient characteristics according to tertiles of serum renin are shown in Table 1. Seven patients with AKI were receiving angiotensin converting enzyme inhibitors or angiotensin receptor blockers at enrollment. Serum renin values were markedly different in the three tertiles: first tertile 7.2 (3.1–12.4) pg/ml, second tertile 40.7 (29.9–60.1) pg/ml, and third tertile 355.3 (180.4–1032.1) pg/ml. While baseline kidney function was similar between the renin tertiles, patients in the highest tertile tended to more often be in the medical ICU, have liver disease, and be more acutely ill as suggested by non-renal APACHE II, non-renal SOFA, number of vasopressors/inotropes, requirement for mechanical ventilation, and have more severe AKI as assessed by serum creatinine and NGAL. The initial measurements of serum renin and NGAL were more than two-fold higher (*p* < 0.001 for both) in patients with AKI compared to those without AKI (Additional file 1: Table S1). At 5–7 days following this initial measurement, both renin (*p* = 0.046) and NGAL (*p* < 0.001) remained higher in patients with AKI versus those without AKI (Additional file 1: Table S1). There was no significant interaction between the first serum renin measurement, AKI status, and the outcome of MAKE (*p* for interaction = 0.251).

**Table 1:** Patient characteristics by renin tertile at first measurement

Patient demographic	Tertile 1 (*n* = 94)	Tertile 2 (*n* = 93)	Tertile 3 (*n* = 93)	*p* value
Serum renin (pg/ml)	7.2 (3.1–12.4)	40.7 (29.9–60.1)	355.3 (180.4–1032.1)	< 0.001
Age (years)	61.5 (47–71)	59 (50–66.5)	53 (40.5–64)	0.004
Sex (% male)	53 (56.4%)	53 (57.0%)	57 (61.3%)	0.760
Race (%)				0.649
White	75 (79.8%)	74 (79.6%)	71 (76.3%)	
Black	10 (10.6%)	9 (9.7%)	7 (7.5%)	
Other	9 (9.6%)	10 (10.8%)	15 (16.1%)	
Weight (kg)	77.7 (65.1–95.3)	88.0 (75.3–105.2)	89.8 (72.8–113.4)	0.006
ICU (%)				0.001
Surgical	46 (48.9%)	37 (39.8%)	18 (19.4%)	
Medical	33 (35.1%)	34 (36.6%)	48 (51.6%)	
Cardiac	15 (16.0%)	22 (23.7%)	27 (29.0%)	
Diabetes (%)	23 (24.5%)	26 (28.0%)	25 (26.9%)	0.858
Hypertension (%)	50 (53.2%)	47 (50.5%)	49 (52.7%)	0.928
Heart failure (%)	20 (21.3%)	20 (21.5%)	31 (33.3%)	0.096
Liver disease (%)	8 (8.5%)	14 (15.1%)	25 (26.9%)	0.003
Cancer (%)	28 (29.8%)	23 (24.7%)	22 (23.9%)	0.613
Baseline serum creatinine (mg/dl)	0.8 (0.7–0.9)	0.8 (0.7–1.0)	0.9 (0.8–1.0)	0.274
Baseline eGFR (ml/min/1.73 m^2^)	89 (78–101)	88 (75–100)	91 (77–105)	0.746
Non-renal APACHE II	15 (10–20)	17 (10.5–22)	19 (12.5–24)	0.009
Non-renal SOFA	5 (2–8)	5 (2–9)	8 (5–12)	< 0.001
Charlson comorbidity index	3 (2–5)	3 (2–4)	4 (2–6)	0.566
Number of vasopressors/inotropes	0 (0–1)	1 (0–2)	2 (1–3)	< 0.001
Mechanical ventilation (%)	44 (46.8%)	47 (50.5%)	62 (66.7%)	0.015
Serum creatinine at first renin measurement (mg/dl)	0.8 (0.7–1.5)	1.3 (0.8–2.4)	1.8 (0.9–3.3)	< 0.001
Acute kidney injury (%)	30 (31.9%)	52 (55.9%)	63 (67.7%)	< 0.001
Stage 2^a^	2 (6.7%)	12 (23.1%)	8 (12.7%)	
Stage 3^a^	28 (93.3%)	40 (76.9%)	55 (87.3%)	
Serum NGAL at first renin measurement (ng/ml)	119 (69–288)	226 (88–421)	240 (80–757)	0.018

*ICU* intensive care unit, *eGFR* estimated glomerular filtration rate, *APACHE II* Acute Physiology and Chronic Health Evaluation II, *SOFA* sequential organ failure assessment, *NGAL* neutrophil gelatinase-associated lipocalin

^a^As percent of all acute kidney injury cases

**Fig. 1:**
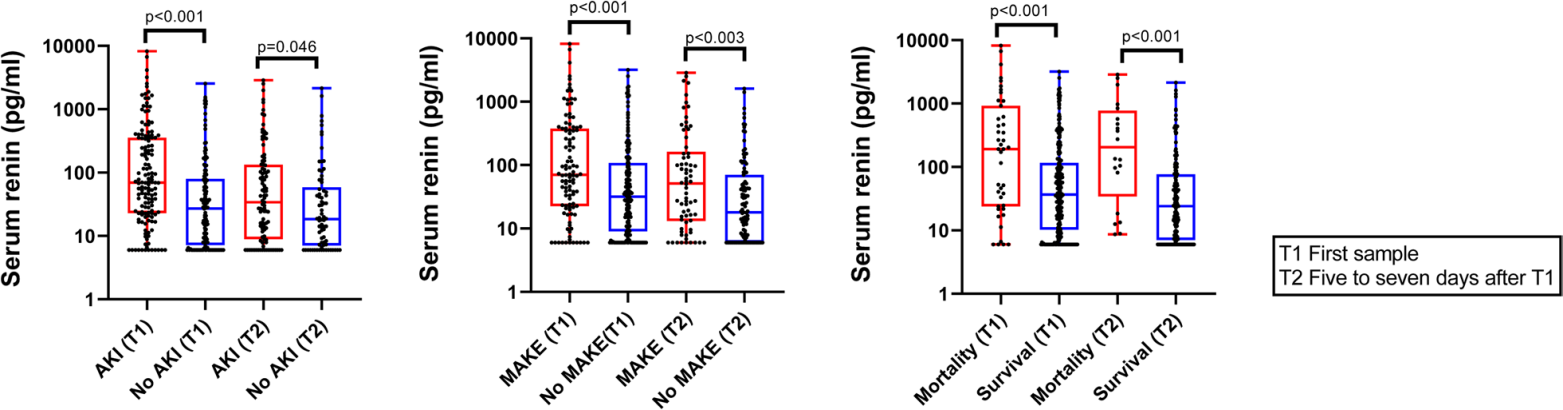
Serum renin stratified by presence of acute kidney injury, major adverse kidney events, or inpatient mortality

For the primary outcome of MAKE assessed at hospital discharge, increasing tertiles of serum renin were strongly associated with a greater frequency of MAKE events (*p* < 0.001) (Table 2). Increasing tertiles of serum renin were also associated with worse secondary outcomes including need for inpatient KRT, duration of mechanical ventilation, and ICU and hospital lengths of stay (*p* ≤ 0.003 for all) (Table 2). Patients experiencing MAKE had significantly higher serum renin and NGAL concentrations at both timepoints (*p* = 0.003 or less for all) (Additional file 1: Table S2). When stratified by inpatient mortality, serum renin and NGAL measurements were again higher at both time points measured in patients who died compared to those who survived (*p* < 0.001 for all) (Additional file 1: Table S3). Serum renin concentrations are shown in Fig. 1.

**Table 2:** Clinical outcomes by renin tertiles

Outcome	Tertile 1 (*n* = 94)	Tertile 2 (*n* = 93)	Tertile 3 (*n* = 93)	*p* value
Primary outcomes				
MAKE at hospital discharge (%)	23 (24.5%)	36 (38.7%)	47 (50.5%)	0.001
Hospital mortality	8 (8.5%)	10 (10.8%)	25 (26.9%)	–
KRT at discharge	1 (1.1%)	3 (3.2%)	4 (4.3%)	–
Discharge eGFR ≤ 75% of baseline	14 (14.9%)	23 (24.7%)	18 (19.4%)	–
Secondary outcomes				
Inpatient KRT (%)	8 (8.5%)	16 (17.2%)	32 (34.4%)	< 0.001
Duration of mechanical ventilation (days)	0 (0–3)	1 (0–6)	2 (1–8)	0.003
ICU length of stay (days)	4 (2–9)	6 (3–11)	8 (4–18)	0.001
Hospital length of stays (days)	9 (5–17)	10 (5–18)	14 (8–22)	0.002

*MAKE* major adverse kidney event, *KRT* kidney replacement therapy, *eGFR* estimated glomerular filtration rate, *ICU* intensive care unit

In the multivariable logistic regression model for MAKE, the second [OR 2.51 (95% CI 1.08–5.80)] and third [OR 2.33 (95% CI 1.01–5.44)] renin tertiles were independently associated with MAKE when compared to the first renin tertile (Table 3). When the same model was analyzed using serum renin as a continuous variable rather than categorization into tertiles, serum renin remained significantly and independently associated with MAKE (OR 1.19 (95% CI 1.01–1.41) for 1-log unit higher serum renin) (Additional file 1: Table S4). In the multivariable logistic regression model for inpatient mortality, the third renin tertile, but not the second, was associated with in-hospital mortality: OR 2.98 (95% CI 1.03–8.61) (Additional file 1: Table S5). When the stepwise logistic regression model was limited to evaluation of MAKE in survivors only (KRT and reduced eGFR), renin was not significantly associated with MAKE (Additional file 1: Table S6). In a sensitivity analysis of the primary model, a 50% reduction in the eGFR threshold for MAKE rather than 25% reduction was evaluated (event numbers for the different definitions shown in Additional file 1: Table S7). Using the 50% eGFR reduction threshold, renin tertiles did not significantly associate with MAKE at discharge (Additional file 1: Table S8).

**Table 3:** Multivariable logistic regression model for major adverse kidney events at the time of hospital discharge by renin tertiles

Variable	Odds ratio with 95% confidence interval^a^	*p* value
Renin tertile (vs. first tertile)		
Second tertile	2.51 (1.08–5.80)	0.032
Third tertile	2.33 (1.01–5.44)	0.050
Age (vs. first quartile)		
Second quartile	0.32 (0.12–0.86)	0.023
Third quartile	0.31 (0.10–0.95)	0.040
Fourth quartile	0.30 (0.08–1.11)	0.071
Sex (male vs. female)	0.63 (0.32–1.25)	0.188
Race (vs. white)		
Black	2.30 (0.74–7.15)	0.150
Other	1.20 (0.39–3.68)	0.748
Baseline eGFR, per 1 ml/min/1.73 m^2^	1.02 (0.99–1.04)	0.173
Charlson comorbidity index, per 1-unit	1.64 (1.35–2.00)	< 0.001
ICU (vs. surgical)		
Medical	2.82 (1.30–6.12)	0.009
Cardiac	2.12 (0.69–6.48)	0.189
Site (UK vs. UTSW)	1.28 (0.54–3.02)	0.572
Non-renal APACHE II, per 1-unit	1.02 (0.98–1.06)	0.246
NGAL, per 1-log unit	2.93 (2.02–4.23)	< 0.001

*eGFR* estimated glomerular filtration rate, *ICU* intensive care unit, *UK* University of Kentucky, *UTSW* University of Texas Southwestern, *APACHE II* Acute Physiology and Chronic Health Evaluation II, *NGAL* neutrophil gelatinase-associated lipocalin

^a^Fixed model developed using hypothesized predictors of outcome and clinically relevant imbalances in renin tertiles

Renin as a continuous variable was further examined in a mixed linear model with repeated measures analysis. The estimates and 95% CI were exponentiated back to geometric means and 95% CI presented. In the group of patients not experiencing MAKE, renin decreased significantly over time: geometric means decreased from 26 to 12 (*p* < 0.001) (Additional file 1: Table S9). In the group of patients experiencing MAKE, renin also decreased over time: geometric means decreased from 81 to 39 (*p* = 0.002). There was no significant difference (no interaction) between groups for renin decrease over time for the outcome of MAKE (*p* = 0.745). In contrast, when analyzing mortality, there was an interaction (*p* = 0.041) between the mortality and survival groups for renin decrease over time (Additional file 1: Table S10). Patients surviving had a decreasing serum renin trend whereas patients that died had similar renin concentrations at the second time point compared to the first measurement.

To investigate the relative contribution of both AKI and hemodynamic instability to elevations in serum renin concentration, a 2 × 2 table with row and column comparisons is shown in Additional file 1: Table S11. The receipt of vasopressors during the ICU admission was associated with elevated renin concentrations measured at the first time point, independent of AKI status. In addition, patients with AKI had higher serum renin concentrations compared to patients without AKI, independent of the need for vasopressors during the ICU admission, although this comparison did not reach statistical significance.

## Discussion

In this multicenter prospective study, serum renin measured within the first days of ICU admission was significantly associated with MAKE. In particular, the association with hospital mortality appears to be the strongest association from the individual components of MAKE. This study adds to the body of literature suggesting that the RAS, at least in the upstream portion represented by serum renin, is activated in critical illness and is associated with mortality and adverse kidney outcomes. The renin measurements in our study, particularly for patients with AKI and those experiencing MAKE, were much higher than their comparators, and at times, 10–100 × fold higher in comparison to renin levels from other patients in the study and the expected normal serum values.

The prior work in this field has primarily focused on critically ill patients with sepsis. In a cohort study of 30 critically ill patients with severe sepsis compared to 10 healthy controls, plasma renin activity (PRA) and angiotensin II were higher at 8- and 24-h following the recognition of organ dysfunction. Furthermore, elevated PRA was negatively correlated with microvascular function as assessed using the reoxygenation rate of skeletal muscle to reactive hyperemia using near infrared spectroscopy [15]. While hypotension is known to elicit renin release, the mean arterial pressure in this study was not significantly correlated with PRA, suggesting that activated RAS in severe sepsis is due to altered explanations, perhaps either causally or even compensatory to microvascular dysfunction and reduced tissue perfusion [15]. Renin is also known to be released in response to sympathetic activation and metabolic alterations, which are common in critically ill patients in the ICU [16].

In a prospective study of 41 patients with septic shock requiring ≥ 0.25 mcg/kg/min of norepinephrine, plasma renin was much higher in patients with KDIGO stage 2 or 3 AKI as compared to KDIGO stage 1 or no AKI [7]. This relationship has been difficult to tease apart as plasma renin is also moderately-strongly correlated with severity of illness scores, vasopressor dose, lactate, and similar markers [7]. Our results demonstrate that even controlling for these severity of illness indicators as well as kidney injury using serum NGAL, serum renin remains associated with MAKE. The challenge of further work in the field remains to ascertain whether elevated renin in the setting of critical illness remains a signal of damage at the microcirculatory level, or if elevated circulatory renin exerts untoward RAS-mediated effects that worsen the cycle of morbidity in AKI and other critical illness syndromes.

The concept of renin as a marker of tissue perfusion was recently tested in a single center study of 20 patients with 112 arterial blood samples and confirmed plasma renin was not affected by diurnal variation, medications, or removal by continuous renal replacement therapy (CRRT) [6]. Similar to our results, the authors demonstrated renin levels with extreme elevation (> 100-fold upper limit of normal) in patients that did not survive. Additionally, renin outperformed lactate for predicting ICU mortality [6]. Further, the value of repeated measures of renin in critical care settings remains unexplored. In our study, we noted that patients whose renin serum concentrations did not decrease by the second time measurement were more likely to die compared to surviving patients whose renin serum concentrations decreased. This difference in trajectories of the serum renin concentration was notable for mortality, but not for MAKE. However, in cardiac surgery patients, the change in serum renin concentrations from pre- and post-operation were recently shown to predict AKI better than the postoperative renin concentration alone [17]. These data all support the potential utility of repeated measures of circulatory renin.

Importantly, the assay technique for serum renin could significantly impact the findings. Previous literature uses both PRA and direct measurement of renin, both of which have trade-offs in their assessment. PRA results may be dependent on angiotensinogen which could be altered by comorbidities or medications in critically ill patients, and also has shown inter-laboratory variability [6, 18]. The measurement of renin in our study is unique from prior literature in this area in that the assay measures and directly quantifies active renin concentrations and shows low cross-reactivity for the precursor of renin, pro-renin [6]. While it has been shown that in stressed states, the renin release far exceeds increases in prorenin and that longer stimuli (i.e. not an acute stimuli) are required to impact prorenin levels, previous work measuring total renin (renin and pro-renin) has been unable to distinguish the specific molecular increase of renin in critical illness [19, 20]. Our results confirm that active renin is elevated, and at times markedly elevated, in those patients with mortality and kidney injury from a broad etiology of critical illness syndromes. Interestingly, the prorenin receptor (PRR) binds both renin and prorenin, and may induce inflammatory and profibrotic gene expression via angiotensin-independent extracellular-signal-regulated kinase 1 (ERK1), ERK2, and p38 mitogen-activated protein kinase (MAPK) signaling pathways [21, 22]. This may provide yet another possible mechanism for renin to function in a causal detrimental pathway in critical illness rather than a marker of illness alone [21, 22].

The statement that the RAS is over-activated in critical illness may, however, be an over-simplification of a complex pathway. In a secondary analysis of the Angiotensin II for the Treatment of High-Output Shock (ATHOS-3) trial enrolling patients with refractory vasodilatory shock, significant ACE deficiency was hypothesized based on elevated angiotensin I: angiotensin II ratios of peptides [2]. Renin concentrations were shown to positively correlate with this angiotensin I: angiotensin II ratio, suggesting that renin could be a surrogate marker in vasodilatory shock for a relative angiotensin II deficiency [23]. Renin concentrations rapidly fell in the group given exogenous angiotensin II, which supports this hypothesis [23]. Similar to our findings, renin was significantly associated with mortality in the ATHOS-3 study [23]. This concept of deficient angiotensin II signaling has been further suggested in recent reports of sepsis-associated AKI [24]. Based on these conflicting reports of RAS over-activation throughout the traditional RAS axis [15], as well as those reviewed here suggesting incomplete activation in the traditional RAS axis, this may be an indication that different RAS endotypes exist in critical illness. If this is indeed the case, measuring renin is much closer to bedside application than the technically challenging measurement of angiotensin peptides [5].

Strengths of this study include its prospective nature, standardized biospecimen collection at two time points, multicenter design, ruling out the influence of chronic kidney disease on the RAS by requirement of documented baseline eGFR ≥ 60 mL/min/1.73 m^2^, careful consideration of covariates known to influence MAKE, measurement of active renin (as opposed to renin and prorenin), and broad generalizability to a range of critically ill patients. To our knowledge, this is the largest study to date evaluating serum or plasma renin concentrations and outcomes in critically ill patients.

Our study also has important limitations. Due to the relatively large number of mortality events contributing to MAKE, we may have been under powered to detect an association with the kidney-specific outcomes in those patients surviving the hospital admission. This limitation is also suspected in the sensitivity analysis for MAKE using a 50% eGFR reduction from baseline where the number of eGFR events contributing to MAKE was reduced by almost half from the primary analysis. We also used a singular race-based eGFR estimate (assessed as ≤ 75% of baseline or not) to adjudicate part of the MAKE outcome. In general, eGFR equations were developed for evaluation of kidney functional status at steady state and are currently under revision by professional societies for their inclusion of race, which may have implications for studies using eGFR as a component of an outcome measure in subacute settings [25]. Importantly, our study did not enroll any patients with KDIGO stage 1 AKI, an important group of patients with potentially reversible kidney injury in clinical practice. However, the latter is also advantageous to eliminate the confounding effect of pre-renal azotemia as intrinsic AKI. While we adjusted for hemodynamic instability with non-renal APACHE II scores in multivariable modeling, as well as investigated the impact of vasopressor receipt while in the ICU on serum renin concentrations in additional analysis, future work would benefit from precise measurements of vasopressor support in order to disentangle the contribution of shock (and vasopressor support) from AKI in terms of elevations in serum renin concentrations in critically ill patients. Additionally, RAS alterations in various diseases such as sepsis and post-operative conditions may be unique, along with potential age and sex differences in the RAS that will be important avenues of future research [26, 27]. While a small proportion of patients were receiving RAS modulating drugs at enrollment, the extent of pre-hospital use of RAS modulating drugs in this cohort is unknown. Even though the medications may have been stopped for several days before biospecimen collection, their impact on the renin response in critical illness is unclear. Finally, given the complexity of biospecimen collection in critically ill patients, renin measurements were not always obtained within the first 3 days of ICU admission, which may have shed additional light on temporal findings of the RAS in early stages of critical illness.

## Conclusions

In a broad cohort of critically ill patients admitted to the ICU, early measurement of serum renin was significantly and independently associated with MAKE assessed at hospital discharge. The association appears to be particularly strong for the relationship between higher serum renin measurements and inpatient mortality. Further studies are needed to elucidate the value of renin as a surrogate pathobiologic marker of microcirculatory function during critical illness.

## Supplementary Information

**Additional file 1**. **Table S1.** Serum Renin and NGAL Measurements in Patients With and Without Acute Kidney Injury. **Table S2.** Serum Renin and NGAL Measurements: MAKE-Discharge vs. Not. **Table S3.** Serum Renin and NGAL Measurements: Survival vs. Death. **Table S4.** Multivariable Regression Model for Major Adverse Kidney Events at the time of Hospital Discharge by Renin as a Continuous Variable. **Table S5.** Stepwise Logistic Regression Model for Inpatient Mortality by Renin Tertiles. **Table S6.** Stepwise Logistic Regression for the Adverse Kidney Outcomes Component of MAKE at Discharge in Survivors. **Table S7.** Event Numbers with 25% vs. 50% eGFR Reduction for MAKE Evaluation. **Table S8.** Sensitivity Analysis of Primary Model Using 50% eGFR Reduction for MAKE. **Table S9.** Repeated Measures Mixed Model for Serum Renin and MAKE at Hospital Discharge. **Table S10.** Repeated Measures Mixed Model for Serum Renin and Hospital Mortality. **Table S11.** Serum Renin Stratified by AKI and ICU Vasopressor Requirement Status.

## Data Availability

Available upon reasonable request to the corresponding author.

## Abbreviations

RAS: Renin-angiotensin system
ACE: Angiotensin converting enzyme
AKI: Acute kidney injury
ICU: Intensive care unit
eGFR: Estimated glomerular filtration rate
ESKD: End stage kidney disease
KDIGO: Kidney Disease: Improving Global Outcomes
APACHE: Acute Physiology and Chronic Health Evaluation
MAKE: Major adverse kidney event
KRT: Kidney replacement therapy
NGAL: Neutrophil gelatinase-associated lipocalin
