# Supplementary material for: Serum renin and major adverse kidney events in critically ill patients: a multicenter prospective study
Source: Crit Care. 2021 Aug 14;25:294. doi: 10.1186/s13054-021-03725-z (PMC8364694; doi:10.1186/s13054-021-03725-z)
Supplement: Supplementary file 1 — Additional file 1. Table S1. Serum Renin and NGAL Measurements in Patients With and Without Acute Kidney Injury. Table S2. Serum Renin and NGAL Measurements: MAKE-Discharge vs. Not. Table S3. Serum Renin and NGAL Measurements: Survival vs. Death. Table S4. Multivariable Regression Model for Major Adverse Kidney Events at the time of Hospital Discharge by Renin as a Continuous Variable. Table S5. Stepwise Logistic Regression Model for Inpatient Mortality by Renin Tertiles. Table S6. Stepwise Logistic Regression for the Adverse Kidney Outcomes Component of MAKE at Discharge in Survivors. Table S7. Event Numbers with 25% vs. 50% eGFR Reduction for MAKE Evaluation. Table S8. Sensitivity Analysis of Primary Model Using 50% eGFR Reduction for MAKE. Table S9. Repeated Measures Mixed Model for Serum Renin and MAKE at Hospital Discharge. Table S10. Repeated Measures Mixed Model for Serum Renin and Hospital Mortality. Table S11. Serum Renin Stratified by AKI and ICU Vasopressor Requirement Status. [file 13054_2021_3725_MOESM1_ESM.docx]

**Supplementary Material**

**Serum Renin and Major Adverse Kidney Events in Critically Ill Patients: A Multicenter Prospective Study**

**Alexander H. Flannery, Pharm.D., Ph.D., Victor Ortiz-Soriano, MD, Xilong Li, Ph.D., MBA, Fabiola G. Gianella, MD, Robert D. Toto, MD, Orson W. Moe, MD,**

**Prasad Devarajan, MD, Stuart L. Goldstein, MD, Javier A. Neyra, MD, MSCS**

**Correspondence:**

Javier A. Neyra, MD, MSCS

Assistant Professor

University of Kentucky Medical Center

800 Rose St, MN668

Lexington, KY 40536

Phone: 859-218-0932

Email: [javier.neyra@uky.edu](mailto:javier.neyra@uky.edu)

**Table S1. Serum Renin and NGAL Measurements in Patients With and Without Acute Kidney Injury**

**Table S2. Serum Renin and NGAL Measurements: MAKE-Discharge vs. Not**

**Table S3. Serum Renin and NGAL Measurements: Survival vs. Death**

**Table S4. Multivariable Regression Model for Major Adverse Kidney Events at the time of Hospital Discharge by Renin as a Continuous Variable**

**Table S5. Stepwise Logistic Regression Model for Inpatient Mortality by Renin Tertiles**

**Table S6. Stepwise Logistic Regression for the Adverse Kidney Outcomes Component of MAKE at Discharge in Survivors**

**Table S7. Event Numbers with 25% vs. 50% eGFR Reduction for MAKE Evaluation**

**Table S8. Sensitivity Analysis of Primary Model Using 50% eGFR Reduction for MAKE**

**Table S9. Repeated Measures Mixed Model for Serum Renin and MAKE at Hospital Discharge**

**Table S10. Repeated Measures Mixed Model for Serum Renin and Hospital Mortality**

**Table S11. Serum Renin Stratified by AKI and ICU Vasopressor Requirement Status**

**Table S1. Serum Renin and NGAL Measurements in Patients With and Without Acute Kidney Injury**

| **Biomarker** | **#** | **AKI (n=145)** | **#** | **No AKI (n=135)** | **p value** |
| --- | --- | --- | --- | --- | --- |
| Renin first measurement (pg/ml) | 145 | 70 (23-355) | 135 | 27 (7-77) | <0.001 |
| NGAL first measurement (ng/ml) | 145 | 411 (206-847) | 135 | 89 (61-133) | <0.001 |
| Renin 5-7 days (pg/ml) | 103 | 34 (9-133) | 74 | 18 (7-59) | 0.046 |
| NGAL 5-7 days (ng/ml) | 103 | 252 (103-524) | 74 | 88 (64-140) | <0.001 |

**Table S2. Serum Renin and NGAL Measurements: MAKE-Discharge vs. Not**

| **Biomarker** | **#** | **MAKE (n=106)** | **#** | **No MAKE (n=174)** | **p value** |
| --- | --- | --- | --- | --- | --- |
| Renin first measurement (pg/ml) | 106 | 71 (22-379) | 174 | 31 (9-99) | <0.001 |
| NGAL first measurement (ng/ml) | 106 | 421 (237-923) | 174 | 104 (66-218) | <0.001 |
| Renin 5-7 days (pg/ml) | 70 | 52 (13-162) | 107 | 18 (4-70) | 0.003 |
| NGAL 5-7 days (ng/ml) | 70 | 302 (123-582) | 107 | 99 (68-211) | <0.001 |

**Table S3. Serum Renin and NGAL Measurements: Survival vs. Death**

| **Biomarker** | **#** | **Death (n=43)** | **#** | **Survival (n=237)** | **p value** |
| --- | --- | --- | --- | --- | --- |
| Renin first measurement (pg/ml) (pg/ml) | 43 | 192 (24-921) | 237 | 36 (10-112) | <0.001 |
| NGAL first measurement (ng/ml) | 43 | 763 (298-1380) | 237 | 129 (72-349) | <0.001 |
| Renin 5-7 days (pg/ml) | 20 | 205 (34-775) | 157 | 24 (7-77) | <0.001 |
| NGAL 5-7 days (ng/ml) | 20 | 687 (188-1045) | 157 | 127 (73-307) | <0.001 |

**Table S4. Multivariable Regression Model for Major Adverse Kidney Events at the time of Hospital Discharge by Renin as a Continuous Variable**

| **Variable** | **Odds Ratio with 95% Confidence Interval** | **p-value** |
| --- | --- | --- |
| Serum renin, per 1-log unit | 1.19 (1.01-1.41) | 0.038 |
| Age (vs. first quartile) |  |  |
| Second Quartile | 0.36 (0.14-0.96) | 0.042 |
| Third Quartile | 0.34 (0.11-1.05) | 0.060 |
| Fourth Quartile | 0.34 (0.09-1.25) | 0.103 |
| Sex (male vs. female) | 0.62 (0.31-1.24) | 0.181 |
| Race (vs. white) |  |  |
| Black | 2.20 (0.71-6.79) | 0.172 |
| Other | 1.17 (0.39-3.56) | 0.780 |
| Baseline eGFR, per 1 ml/min/1.73m^2^ | 1.02 (0.99-1.04) | 0.153 |
| Charlson Comorbidity Index, per 1-unit | 1.62 (1.33-1.98) | <0.001 |
| ICU (vs. surgical) |  |  |
| Medical | 2.68 (1.24-5.81) | 0.012 |
| Cardiac | 2.01 (0.66-6.10) | 0.218 |
| Site (UK vs. UTSW) | 1.28 (0.54-3.01) | 0.573 |
| Non-renal APACHE II, per 1-unit | 1.02 (0.99-1.06) | 0.230 |
| NGAL, per 1-log unit | 2.86 (1.98-4.12) | <0.001 |

^a^Fixed model developed using hypothesized predictors of outcome and clinically relevant imbalances in renin tertiles

eGFR=estimated glomerular filtration rate; ICU=intensive care unit; UK=University of Kentucky; UTSW=University of Texas Southwestern; APACHE II= Acute Physiology and Chronic Health Evaluation II; NGAL= neutrophil gelatinase-associated lipocalin

**Table S5. Stepwise Logistic Regression Model for Inpatient Mortality by Renin Tertiles**

| **Variable** | **Odds Ratio with 95% Confidence Interval^a^** | **p-value** |
| --- | --- | --- |
| Renin Tertile (vs. first tertile) |  |  |
| Second Tertile | 1.18 (0.37-3.74) | 0.783 |
| Third Tertile | 2.98 (1.03-8.61) | 0.044 |
| Age (vs. first quartile) |  |  |
| Second quartile | 0.46 (0.14-1.57) | 0.215 |
| Third quartile | 0.22 (0.06-0.88) | 0.032 |
| Fourth Quartile | 0.91 (0.25-3.25) | 0.879 |
| Race (vs. white) |  |  |
| Black | 3.10 (0.82-11.8) | 0.097 |
| Other | 3.05 (0.80-11.6) | 0.101 |
| Charlson Comorbidity Index, per 1-unit | 1.29 (1.09-1.54) | 0.004 |
| Non-renal APACHE II, per 1-unit | 1.04 (1.01-1.07) | 0.019 |
| NGAL, per 1-log unit | 3.23 (2.10-4.97) | <0.001 |

^a^backward stepwise regression (p<0.2 threshold)

APACHE II= Acute Physiology and Chronic Health Evaluation II; NGAL= neutrophil gelatinase-associated lipocalin

**Table S6.** **Stepwise Logistic Regression for the Adverse Kidney Outcomes Component of MAKE at Discharge in Survivors**

| **Variable** | **Odds Ratio with 95% Confidence Interval^a^** | **p-value** |
| --- | --- | --- |
| Renin Tertile (vs. first tertile) |  |  |
| Second Tertile | 2.29 (0.94-5.59) | 0.070 |
| Third Tertile | 1.51 (0.59-3.84) | 0.385 |
| Age (vs. first quartile) |  |  |
| Second quartile | 0.26 (0.09-0.75) | 0.013 |
| Third quartile | 0.30 (0.10-0.83) | 0.025 |
| Fourth Quartile | 0.15 (0.04-0.54) | 0.004 |
| Charlson Comorbidity Index, per 1-unit | 1.61 (1.32-1.97) | <0.001 |
| ICU (vs. surgical) |  |  |
| Medical | 3.87 (1.62-9.24) | 0.002 |
| Cardiac | 2.72 (0.97-7.59) | 0.057 |
| NGAL, per 1-log unit | 2.53 (1.72-3.74) | <0.001 |

^a^backward stepwise regression (p<0.2 threshold)

ICU=intensive care unit; NGAL= NGAL= neutrophil gelatinase-associated lipocalin

**Table S7. Event Numbers with 25% vs. 50% eGFR Reduction for MAKE Evaluation**

| **MAKE Definitions** | **Total events (n)** |
| --- | --- |
| **MAKE 25** | 106 |
| Hospital Mortality | 43 |
| KRT at Discharge | 8 |
| Discharge eGFR ≤ 75% of baseline | 55 |
|  |  |
| **MAKE 50** | 81 |
| Hospital Mortality | 43 |
| KRT at Discharge | 8 |
| Discharge eGFR ≤ 50% of baseline | 30 |

**Table S8. Sensitivity Analysis of Primary Model Using 50% eGFR Reduction for MAKE**

| **Variable** | **Odds Ratio with 95% Confidence Interval^a^** | **p-value** |
| --- | --- | --- |
| Renin Tertile (vs. first tertile) |  |  |
| Second Tertile | 2.01 (0.87-4.64) | 0.100 |
| Third Tertile | 1.82 (0.78-4.23) | 0.166 |
| Age (vs. first quartile) |  |  |
| Second Quartile | 0.29 (0.11-0.81) | 0.017 |
| Third Quartile | 0.44 (0.14-1.34) | 0.147 |
| Fourth Quartile | 0.49 (0.14-1.73) | 0.271 |
| Sex (male vs. female) | 0.86 (0.43-1.72) | 0.675 |
| Race (vs. white) |  |  |
| Black | 1.44 (0.47-4.34) | 0.523 |
| Other | 0.90 (0.27-2.92) | 0.854 |
| Baseline eGFR, per 1 ml/min/1.73m^2^ | 1.01 (0.99-1.03) | 0.340 |
| Charlson Comorbidity Index, per 1-unit | 1.32 (1.11-1.57) | 0.001 |
| ICU (vs. surgical) |  |  |
| Medical | 1.69 (0.79-3.64) | 0.178 |
| Cardiac | 0.93 (0.31-2.82) | 0.901 |
| Site (UK vs. UTSW) | 0.72 (0.31-1.66) | 0.436 |
| Non-renal APACHE II, per 1-unit | 1.02 (0.99-1.05) | 0.319 |
| NGAL, per 1-log unit | 2.83 (1.98-4.04) | <0.001 |

^a^Fixed model developed using hypothesized predictors of outcome and clinically relevant imbalances in renin tertiles

eGFR=estimated glomerular filtration rate; ICU=intensive care unit; UK=University of Kentucky; UTSW=University of Texas Southwestern; APACHE II= Acute Physiology and Chronic Health Evaluation II; NGAL= neutrophil gelatinase-associated lipocalin

**Table S9. Repeated Measures Mixed Model for Serum Renin and MAKE at Hospital Discharge**

| **MAKE-Discharge Outcome** | **Time**  **#** | **Geometric Mean (95% CI)** | **MAKE-Discharge Time 1 vs. Time 2** | **No MAKE at Discharge Time 1 vs. Time 2** | | **MAKE-Discharge vs. No MAKE Time 1** | | **MAKE-Discharge vs. No MAKE Time 2** | | **Interaction** | |
| --- | --- | --- | --- | --- | --- | --- | --- | --- | --- | --- | --- |
| No | 1 | 26 (19-37) | p=0.002 | p<0.001 | p<0.001 | | p<0.001 | | p=0.745 | |  |
| No | 2 | 12 (8-18) |  |  |  | |  | |  | |  |
| Yes | 1 | 81 (54-123) |  |  |  | |  | |  | |  |
| Yes | 2 | 39 (23-67) |  |  |  | |  | |  | |  |

**Table S10. Repeated Measures Mixed Model for Serum Renin and Hospital Mortality**

| **Mortality Outcome** | **Time**  **#** | **Geometric Mean (95% CI)** | **Mortality Time 1 vs. Time 2** | **Survived Time 1 vs. Time 2** | | **Mortality vs. Survival Time 1** | | **Mortality vs. Survival Time 2** | | **Interaction** | |
| --- | --- | --- | --- | --- | --- | --- | --- | --- | --- | --- | --- |
| No | 1 | 33 (25-43) | p=0.864 | p<0.001 | p<0.001 | | p<0.001 | | p=0.041 | |  |
| No | 2 | 14 (10-20) |  |  |  | |  | |  | |  |
| Yes | 1 | 129 (67-248) |  |  |  | |  | |  | |  |
| Yes | 2 | 139 (54-358) |  |  |  | |  | |  | |  |

**Table S11. Serum Renin Stratified by AKI and ICU Vasopressor Requirement Status**

|  | **Patients with AKI** | **Patients without AKI** | **P-value for Column Comparisons** |
| --- | --- | --- | --- |
| **Received Vasopressors in ICU** | **n=115** | **n=42** |  |
| Serum renin (pg/ml) | 86.1 (30.3-391.1) | 47.9 (11.9-220) | 0.054 |
|  |  |  |  |
| **P-value for Row Comparisons** | 0.002 | 0.001 |  |
|  |  |  |  |
| **No Receipt of Vasopressors in ICU** | **n=30** | **n=93** | 0.074 |
| Serum renin (pg/ml) | 24.0 (9.7-81.4) | 16.7 (5.3-51.4) |  |
